# Single Muscle Fiber Gene Expression with Run Taper

**DOI:** 10.1371/journal.pone.0108547

**Published:** 2014-09-30

**Authors:** Kevin Murach, Ulrika Raue, Brittany Wilkerson, Kiril Minchev, Bozena Jemiolo, James Bagley, Nicholas Luden, Scott Trappe

**Affiliations:** Human Performance Laboratory, Ball State University, Muncie, Indiana, United States of America; West Virginia University School of Medicine, United States of America

## Abstract

This study evaluated gene expression changes in gastrocnemius slow-twitch myosin heavy chain I (MHC I) and fast-twitch (MHC IIa) muscle fibers of collegiate cross-country runners (n = 6, 20±1 y, VO_2max_ = 70±1 ml•kg^−1^•min^−1^) during two distinct training phases. In a controlled environment, runners performed identical 8 kilometer runs (30∶18±0∶30 min:s, 89±1% HR_max_) while in heavy training (∼72 km/wk) and following a 3 wk taper. Training volume during the taper leading into peak competition was reduced ∼50% which resulted in improved race times and greater cross-section and improved function of MHC IIa fibers. Single muscle fibers were isolated from pre and 4 hour post run biopsies in heavily trained and tapered states to examine the dynamic acute exercise response of the growth-related genes Fibroblast growth factor-inducible 14 (*FN14*), Myostatin (*MSTN*), Heat shock protein 72 (*HSP72*), Muscle ring-finger protein-1 (*MURF1*), Myogenic factor 6 (*MRF4*), and Insulin-like growth factor 1 (*IGF1*) via qPCR. *FN14* increased 4.3-fold in MHC IIa fibers with exercise in the tapered state (P<0.05). *MSTN* was suppressed with exercise in both fiber types and training states (P<0.05) while *MURF1* and *HSP72* responded to running in MHC IIa and I fibers, respectively, regardless of training state (P<0.05). Robust induction of *FN14* (previously shown to strongly correlate with hypertrophy) and greater overall transcriptional flexibility with exercise in the tapered state provides an initial molecular basis for fast-twitch muscle fiber performance gains previously observed after taper in competitive endurance athletes.

## Introduction

Athletes routinely reduce training volume prior to a major competition to help facilitate peak performance. A 3–4 week reduced training phase, known as taper, typically results in a 2–4% improvement in race performance [1]–[3]. In the last 30 years, taper research has identified increased power output at the whole muscle and single muscle fiber level as an important physiological basis for improved performance [1], [2], [4]. More specifically, tapering has been repeatedly shown to target fast-twitch muscle fibers by increasing their size [1]–[3] and power [1], [2] with improvements in contractile performance largely accounted for by hypertrophy. However, little is known about molecular alterations that are contributing to performance gains in fast-twitch muscle fibers with tapering.

Recent methodological advances in our laboratory have established the ability to examine gene expression at the single muscle fiber level [5]–[7]. We sought to apply this novel approach with tapering to better understand potential molecular adaptations in fast-twitch muscle fibers. We were guided into these single muscle fiber gene studies by previous work in cross-country runners (for whom muscle biopsy samples were still available) that had an altered transcriptional response in mixed-muscle homogenate samples after identical 8 km running bouts in the heavily trained versus tapered state [2]. This alteration was intriguing since previous research has shown a blunted transcriptional response to exercise in well-conditioned skeletal muscle [6], [8]. Thus, it appears that skeletal muscle of highly trained athletes may be more sensitive at the molecular level to various training phases than previously thought. Further support for conducting these single fiber gene studies was that the runners’ MHC IIa fibers hypertrophied (+15%) and increased power output (+9%) with taper [2]. The combination of single muscle fiber gene measurements at strategically timed muscle biopsies after a heavily trained and tapered state 8 km run provided a unique opportunity to gauge the transcriptional flexibility of the MHC IIa fibers during these two training periods.

For the single muscle fiber gene experiments, we selected six genes that have been implicated in muscle size regulation and remodeling and included *FN14*, *MSTN*, *HSP72*, *MURF1*, *MRF4*, and *IGF1*. In developed human skeletal muscle, Fibroblast growth factor-inducible 14 (*FN14/TNFRSF12A*) was recently shown to strongly correlate with fast-twitch specific growth in response to exercise [6], [9], the cytokines Myostatin (*MSTN/GDF8*) and Insulin-like growth factor 1 (*IGF1*) are components of major growth pathways [10], Myogenic factor 6 (*MRF4/MYF6*) is associated with exercise-induced remodeling [7], Muscle ring-finger protein-1 (*MURF1/TRIM63*) is a marker of ubiquitin-proteasome mediated myofibrillar breakdown [11], and Heat shock protein 72 (*HSP72/HSPA1A*) combats protein degradation [12]. We hypothesized that advantageous post exercise regulation of *HSP72*, *MURF1*, and *MRF4* previously observed in mixed-muscle with taper [2] would be more pronounced in MHC IIa fibers and that favorable *FN14*, *MSTN*, and *IGF1* expression would be targeted to the MHC IIa muscle fibers.

## Materials and Methods

### Subjects

**Figure 1:**
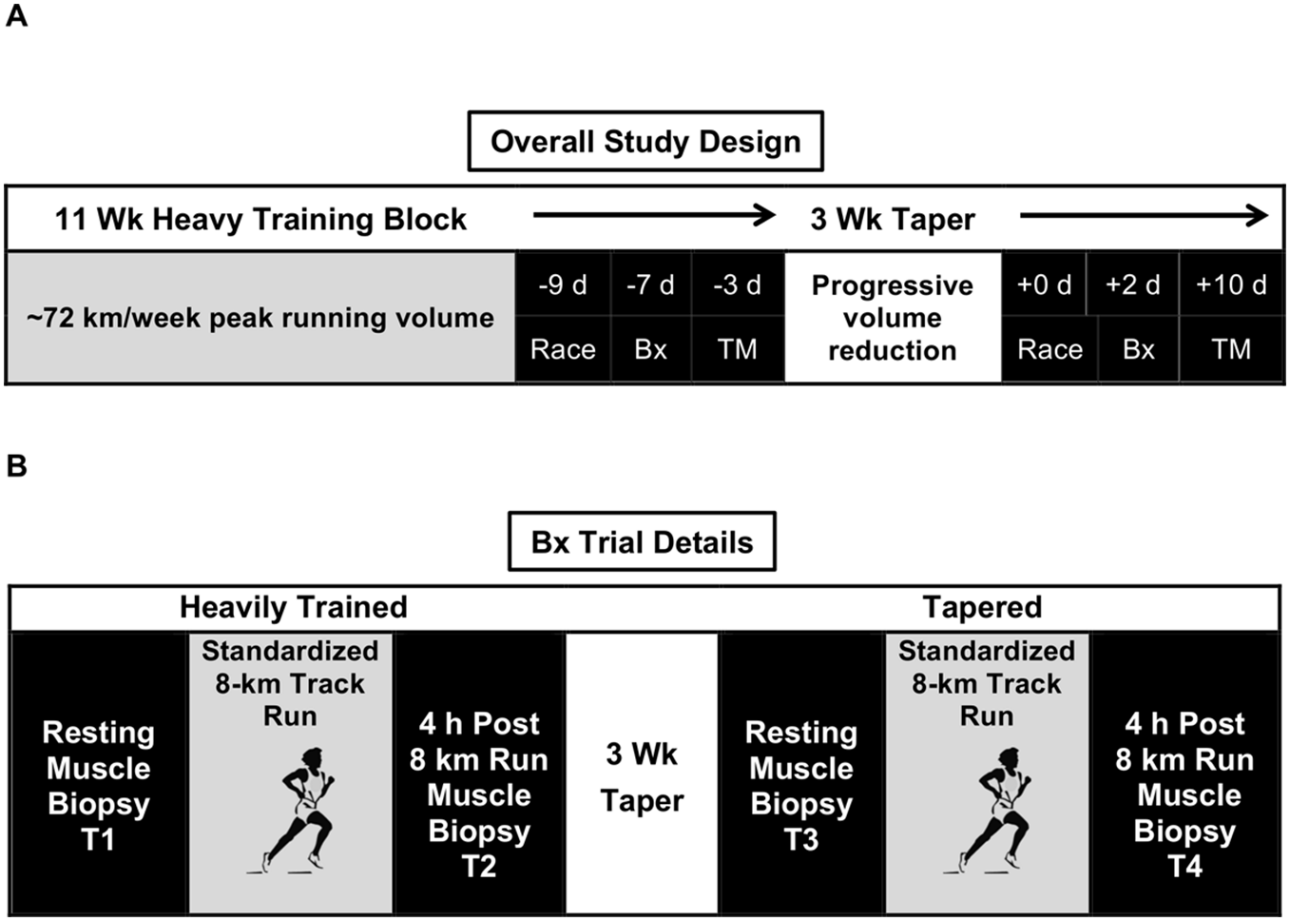
Overall study design (A), and Biopsy trial details (B). Bx = lateral gastrocnemius muscle biopsy, TM = Treadmill VO_2max_ testing.

**Figure 2:**
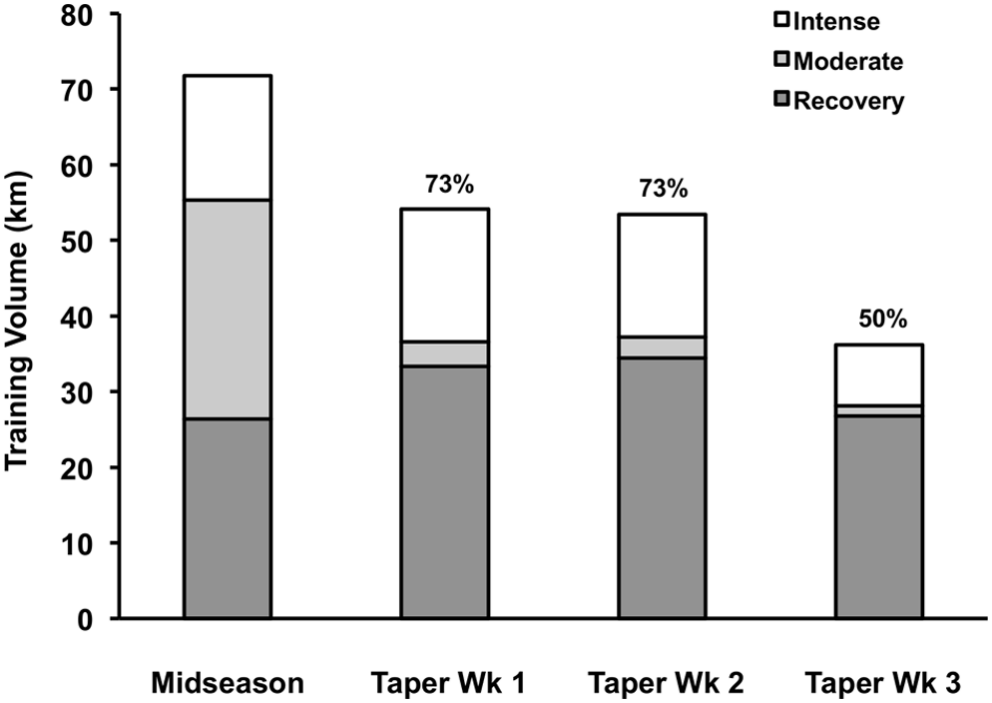
Summary of training volume and intensity during heavy training (11 wks) and each week of taper (3 wks), reproduced from Luden et al. [2] with permission from the American Physiological Society.

Seven male runners from Taylor University’s (Upland, Indiana) cross-country team volunteered to participate in a previous study [2]. Of these seven subjects, six were used for this investigation (age [y] 20±1, height [cm] 178±5, weight [kg] 66.1±5.1) (Table 1) as insufficient tissue at one time point for one subject negated molecular analysis. Details of the general study design and taper program are outlined in our previous investigation [2] and briefly profiled here (Figures 1 and 2). Subjects were competitive runners with 8 km lifetime best average performances of 26∶32±0∶32 (min:s). Prior to the investigation, subjects competitively participated in running for ∼7 years (range: 4.5–10.0 y). Subjects were provided with written and oral information about experimental procedures and potential risks prior to providing written consent.

**Table 1:** Subject characteristics and physiological data (n = 6).

	Heavily Trained	Tapered	% Δ
**MHC IIa CSA (µm^2^)**	5812±622	6631±758	+14%
**MHC IIa Power (µN•FL^−1^•s^−1^)**	58.7±6.4	63.5±7.3	+9%
**VO_2max_ (ml•kg^−1^•min^−1^)**	70.0±1.1	69.1±1.1	↔
**X-Country Race Performance (min:s)**	27∶42±0∶25	27∶00±0∶30	−3%

Data derived from Luden et al. [2] less one subject.

### Ethics Statement

Support was granted by the coaching staff and all procedures were approved by the Ball State University and Taylor University Institutional Review Boards.

### Experimental Design

**Figure 3:**
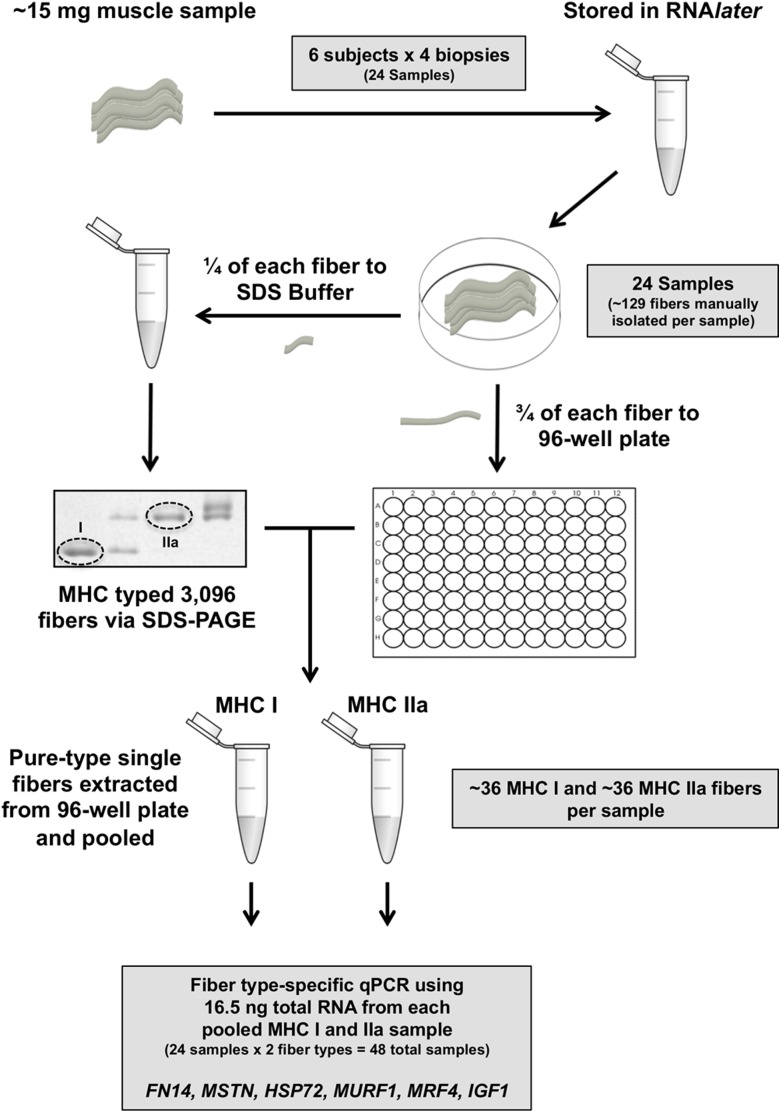
Schematic of method used to conduct fiber type-specific isolation of single muscle fibers for qPCR analysis.

A schematic of the study design is presented in Figure 3. Identical laboratory procedures were performed in the heavily trained (T1 and T2) and tapered (T3 and T4) conditions. Resting (heavily trained - T1, tapered - T3) and 4 h post exercise (heavily trained - T2, tapered - T4) gastrocnemius muscle biopsies were collected around an 8 km standardized indoor track run. MHC I and IIa fibers were individually isolated and pooled in order to conduct targeted fiber type specific gene expression analysis via reverse transcription quantitative real-time polymerase chain reaction (qPCR). The effect of taper was examined by conducting exercise response comparisons (T1:T2 and T3:T4) in both fiber types. Subjects were treadmill tested for aerobic capacity before and after the taper period.

### Training

Taylor University coaching staff prescribed the training performed throughout the 11 wk season (8 wks of midseason heavy training +3 wks of reduced-volume taper) based upon recommendations from our laboratory team. Training load was quantified using heart rate monitor data (Polar, Lake Success, NY) and self-reported running logs while mid-season training load (8 wks) was aggregated to represent one mean for heavy training. Average mid-season weekly running volume (6 d/wk) was approximately 72 km. During the 3 wk taper, weekly running volume in the moderate intensity range was progressively reduced to 50% of mid-season training while high intensity training volume was maintained (Figure 2).

### Standardized 8 km Indoor Track Run

Subjects were instructed to run 8 km on a 200 m indoor track between 15.2 and 16.0 km/h (∼45–48 s/lap) depending on talent level (30∶18±0∶30 min:s, 89±1% HR_max_). This pace was chosen because it corresponded to a training velocity commonly performed by this group. Lap times were verbalized throughout the run to ensure even pacing and each lap split was recorded manually. Subjects performed identical runs in the heavily trained and tapered state and were monitored by the investigative team.

### Muscle Biopsy

Four muscle biopsies (T1, T2, T3, T4) were obtained from the lateral head of the gastrocnemius. The gastrocnemius was chosen based on its documented use during running [13] and the large amount of gastrocnemius research conducted in runners over the past 40 years. Subjects reported to Taylor University having refrained from physical activity for two days (∼48 h). After 30 min of supine rest, a resting muscle biopsy was obtained (T1, T3) followed by a standardized 8 km run on a 200 m indoor track. Subjects then underwent 4 h of supine rest upon which a second biopsy was obtained from the opposite leg (T2, T4). The rationale for the 4 h post exercise biopsy time point was based on previous post exercise mRNA time course investigations from our laboratory [7], [14]. From each biopsy, a muscle sample weighing ∼15 mg was placed in 0.5 ml of RNA*later* (Ambion, Austin, TX), stored at 4°C overnight, and subsequently stored at −20°C until fiber separation and RNA extraction.

### Muscle Fiber Separation and MHC Identification

See Figure 3 for a schematic representation of the method used for fiber type-specific isolation and fiber pooling for qPCR. Individual muscle fibers were isolated in RNAlater using fine tweezers under a light microscope at room temperature as previously described [5], [11], [15], [16]. An average of 129 fibers were isolated from each of the 24 samples (6 subjects×4 biopsies) for a total of 3,096 fibers. Approximately one quarter of each fiber was clipped and placed into 40 µl sodium dodecyl sulfate (SDS) buffer (1% SDS, 6 mg/ml ethylenediaminetetraacetic acid [EDTA], 0.06 M Tris [pH 6.8], 2 mg/ml bromophenol blue, 15% glycerol, and 5% b-mercaptoethanol) for SDS-PAGE (sodium dodecyl sulfate polyacrylamide gel electrophoresis) MHC identification as previously described by our laboratory [5], [11], [15], [16]. The remaining fiber segment was placed into an individual well of a number-labeled 96-well plate with 75 µl RNA*later* and stored at −20°C until MHC type was confirmed and before fiber type-specific pooling. Overall MHC fiber type distribution of each subject’s lateral gastrocnemius was determined using all fibers extracted from each individual.

### Fiber Pooling and RNA Extraction

Following MHC isoform identification, the corresponding MHC I and IIa fiber segments were located in the 96-well plate. Muscle fibers of each type (MHC I and IIa) at each time point (T1, T2, T3, T4) were extracted from their individual wells containing RNA*later* and combined in a tube containing 500 µl TRI Reagent (Molecular Research Center, Cincinnati OH) used for RNA extraction. RNA extraction was performed according to the manufacturers protocol (MRC) and total RNA concentration (4.07±0.15 ng/µl) was determined using the Quant-iT RNA assay kit (Invitrogen, Carlsbad, CA) in conjunction with the Qubit fluorometer (Invitrogen). Forty-eight samples (24 samples×2 fiber types) were ultimately generated containing 36±6 (range 23–54) fibers, an amount sufficient to yield an RNA concentration of >1.5 ng/µl [6], [7].

### Reverse Transcription and qPCR

Oligo (dT) primed first-strand cDNA was synthesized (16.5 ng of total RNA) using SuperScript II RT (Invitrogen) and quantification of mRNA levels (in duplicate) was performed in a 72-well Rotor-Gene 3000 Centrifugal Real-Time Cycler (Corbett Research, Mortlake, NSW, Australia). Ribosomal protein, large, P0 (*RPLP0*) was used as a reference gene as we have previously described [17]. All primers used in this study were mRNA-specific (on different exons and crossing over an intron) and designed for qPCR (Vector NTI Advance 9 software, Invitrogen) using SYBR Green chemistry. The primer sequences for *FN14* were: Forward -ACTTCTGCCTGGGCTGCGCT and Reverse - TCTCCTGCGGCATCGTCTCC, Genbank number: NM_016639.2. Primer sequences and characteristics for *MSTN*, *HSP72*, *MURF1*, *MRF4*, and *IGF1* as well as qPCR parameters have been reported previously from our laboratory [2], [5], [7], [14], [18]. For each qPCR run, a melting curve analysis was generated to validate the presence of only one product and a serial dilution curve (cDNA made of 500 ng total RNA from human skeletal muscle; Ambion, Austin, TX) was generated to evaluate reaction efficiencies. The amplification calculated by the Rotor-Gene software was specific and highly efficient (efficiency = 1.03±0.02; R^2^ = 0.99±0.00; slope = 3.24±0.04). The gene expression response to exercise (8 km run) was examined in the heavily trained and tapered state using the 2^−ΔΔC_T_^ (fold change) quantification method [14], [19], [20].

### Statistics

Data were checked for normality and sphericity and original or log transformed data were used for statistical analyses. Within each fiber type, the gene expression response to exercise in the heavily trained and tapered state was evaluated using a repeated measures two-way ANOVA with factors of time (T1–T4) and training state (heavily trained and tapered). Due to the minimal number of hybrids (<8%) and no IIx fibers in these highly trained runners, gene expression analysis was limited to MHC I and MHC IIa fibers. All statistics were conducted in SPSS 17.0 for Windows, all data are presented as means ± SE, and significance was set at P<0.05.

## Results

### Exercise-Induced (T1:T2 and T3:T4) Gene Expression in MHC IIa fibers

**Figure 4:**
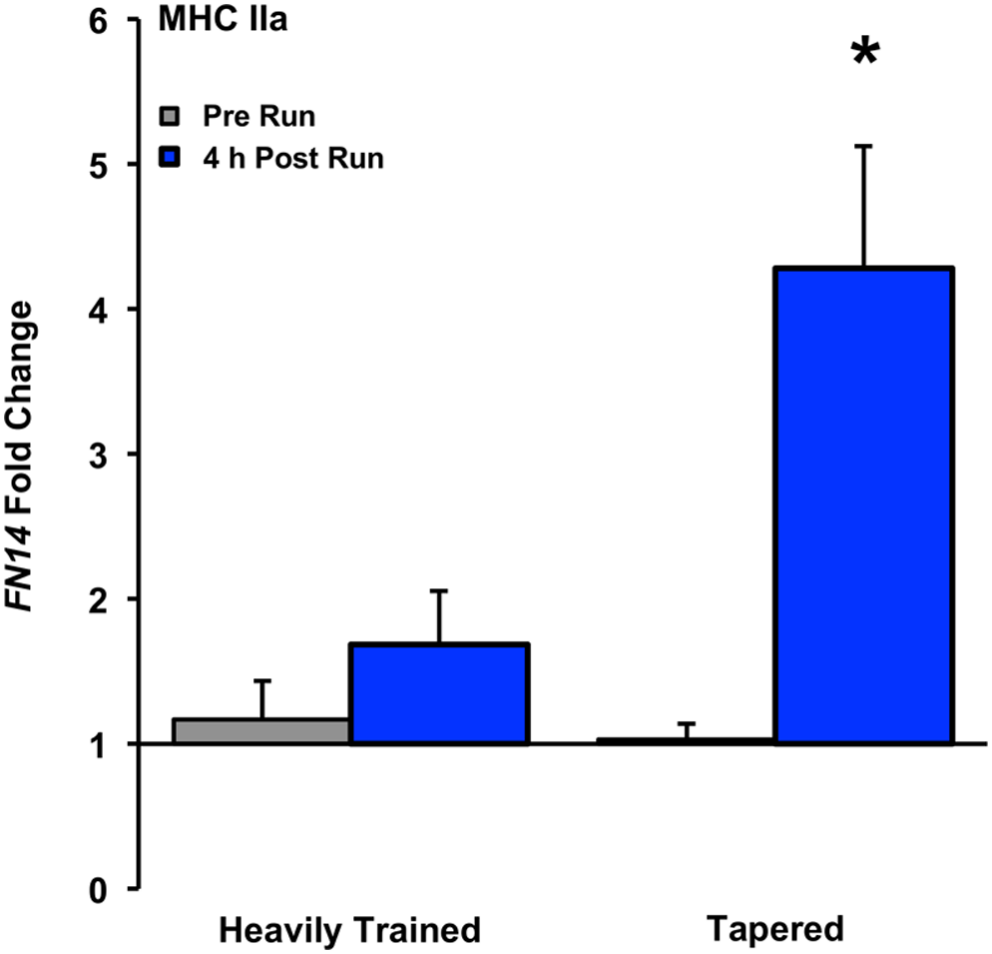
*FN14* gene expression response after an 8 km run in the heavily trained (T1:T2) and tapered (T3:T4) state in MHC IIa fibers, presented as fold change, *Time and interaction effect, P<0.05.

**Figure 5:**
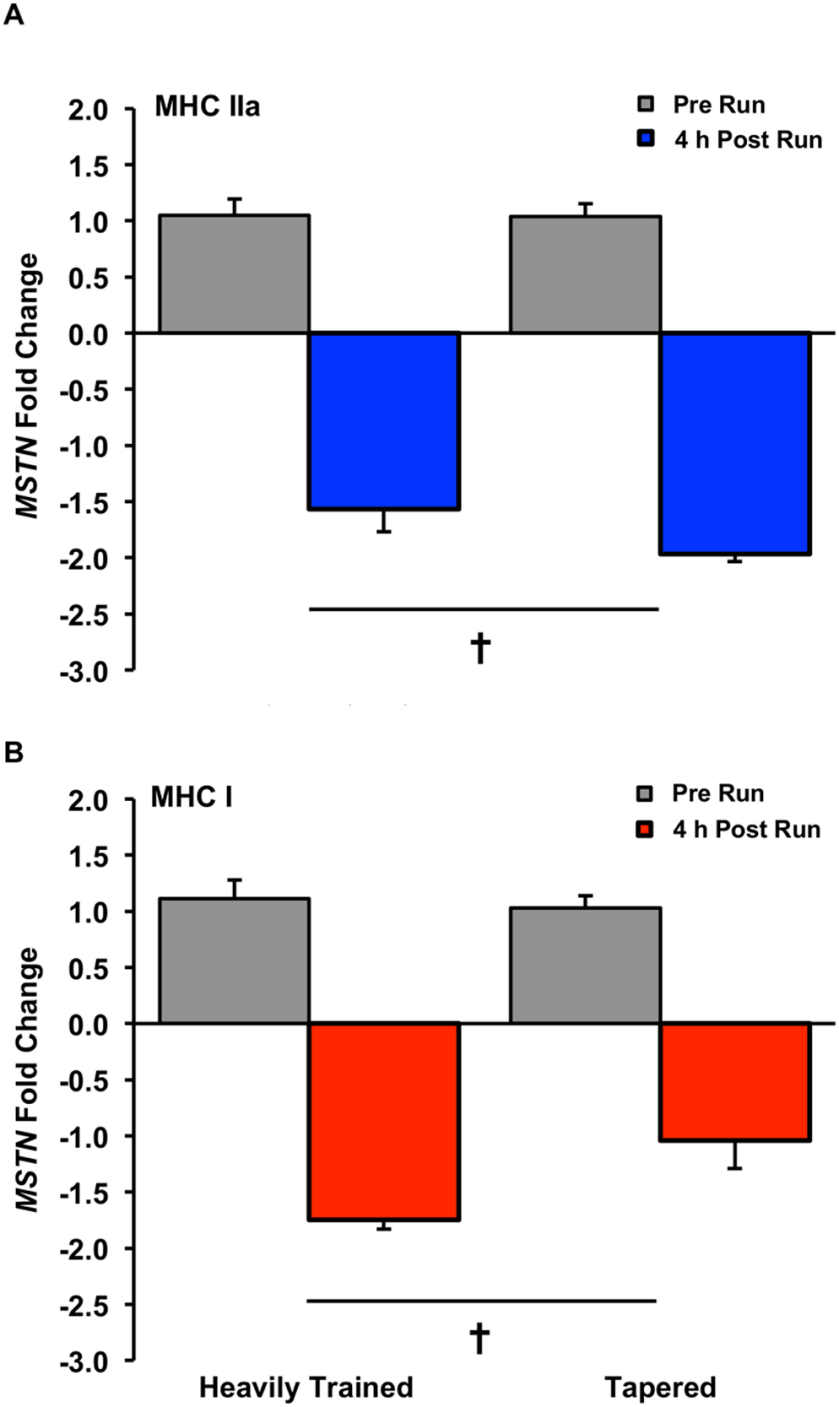
*MSTN* gene expression response after an 8 km run in the heavily trained (T1:T2) and tapered (T3:T4) state in MHC I (A) and IIa (B) fibers, presented as fold change, Main time effect for exercise, P<0.05.

**Figure 6:**
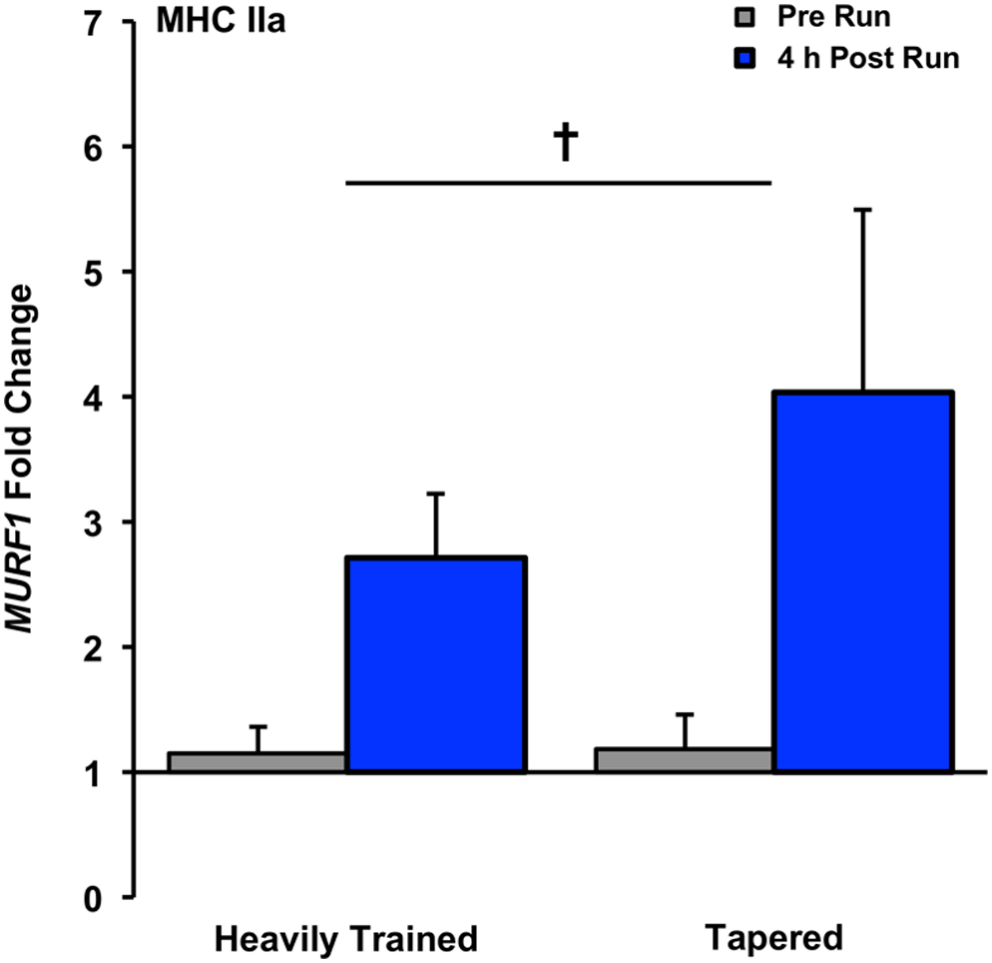
*MURF1* gene expression response after an 8 km run in the heavily trained (T1:T2) and tapered (T3:T4) state in MHC IIa fibers, presented as fold change, Main time effect for exercise, P<0.05.

In the tapered state (T3:T4), *FN14* mRNA increased 4.3-fold (time and interaction effect, P<0.05, Figure 4) 4 h following the 8 km run. *MSTN* decreased 1.6-fold in the heavily trained state and 2.0-fold in the tapered state (time effect, P<0.05, Figure 5A). *MURF1* increased 2.7-fold in the heavily trained state and 4.0-fold in the tapered state (time effect, P<0.05, Figure 6). No changes were observed with exercise for *FN14* in the heavily trained state or *HSP72*, *MRF4*, or *IGF1* in the heavily trained or tapered state (Table 2).

**Table 2:** Genes not responsive to exercise in MHC IIa fibers in the heavily trained or tapered state.

	Heavily Trained		Tapered	
	T1	T2	T3	T4
***HSP72***	1.07±0.17	1.31±0.32	1.06±0.15	1.77±0.32
***MRF4***	1.03±0.12	0.84±0.09	1.15±0.28	0.9±0.11
***IGF1***	1.09±0.17	1.02±0.21	1.18±0.30	0.77±0.12

Presented as fold change, mean ± SE.

### Exercise-Induced (T1:T2 and T3:T4) Gene Expression in MHC I fibers

**Figure 7:**
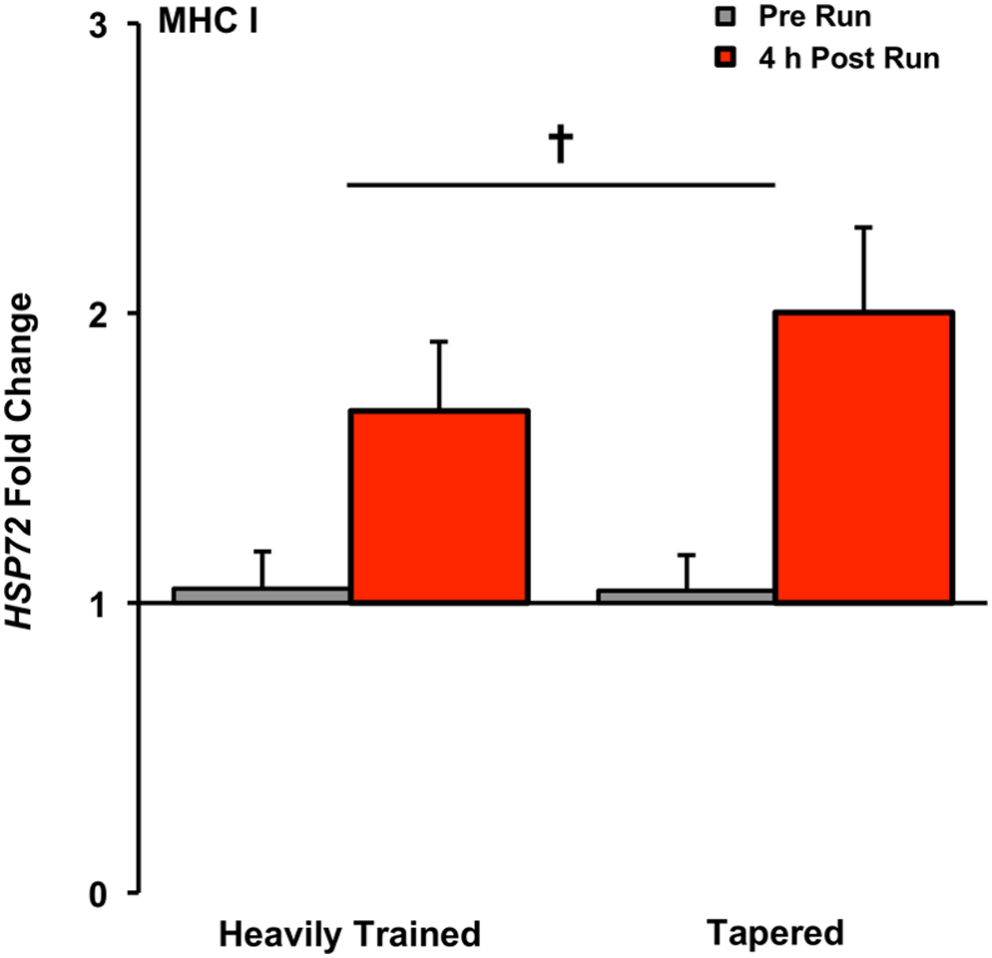
*HSP72* gene expression response after an 8 km run in the heavily trained (T1:T2) and tapered (T3:T4) state in MHC I fibers, presented as fold change, Main time effect for exercise, P<0.05.

Exercise decreased *MSTN* 1.7-fold in the heavily trained state and 1.1-fold in the tapered state (time effect, P<0.05, Figure 5B). *HSP72* increased 1.7-fold in the heavily trained state and 2.0-fold in the tapered state (time effect, P<0.05, Figure 7). No changes occurred in *FN14*, *MURF1*, *MRF4*, or *IGF1* with exercise in the heavily trained or tapered state (Table 3).

**Table 3:** Genes not responsive to exercise in MHC I fibers in the heavily trained or tapered state.

	Heavily Trained		Tapered	
	T1	T2	T3	T4
***FN14***	2.04±1.14	3.18±0.92	1.47±0.53	2.25±1.13
***MURF1***	1.12±0.19	3.94±1.88	1.17±0.29	1.04±0.27
***MRF4***	1.05±0.16	1.45±0.17	1.25±0.41	1.09±0.12
***IGF1***	1.09±0.19	1.03±0.13	1.06±0.17	0.77±0.06

Presented as fold change, mean ± SE.

### Lateral Gastrocnemius Fiber Type

The average fiber type distribution (percentage) of the gastrocnemius in these runners was: 61.7±4.0% MHC I, 4.5±1.6% MHC I/IIa, 30.9±3.1% MHC IIa, 2.7±1.7% MHC IIa/IIx, and 0.2±0.1% MHC I/IIa/IIx. There were no MHC IIx fibers found in any subject. Fibers expressing multiple isoforms (MHC I/IIa, MHC IIa/IIx, and MHC I/IIa/IIx) comprised 7.4% of the total fiber population.

## Discussion

The unique aspect of this investigation was the examination of MHC I and IIa gene responses in competitive collegiate runners after identical 8 km runs in the heavily trained versus tapered state. The 8 km running stimulus used in our study design reflects a typical weekly training run (∼89% HR_max_) these athletes performed throughout the competitive season. The post 4 h run time point coincides with anabolic and catabolic mRNA expression patterns following exercise [7], [14] and captures elements of muscle remodeling. The main finding was that *FN14*, a gene strongly associated with fast-twitch hypertrophy [6], [9], was robustly induced in MHC IIa fibers with exercise in the tapered state. Additionally, *MSTN* was suppressed with exercise in both fiber types and training states while *MURF1* and *HSP72* responded to running in MHC IIa and I fibers, respectively, regardless of training state. Contrary to the notion that exercise gene response [6], [8], [21], [22] and adaptive potential [23] are attenuated as training status improves, these data indicate transcriptional flexibility in MHC I and IIa fibers of highly trained runners after an effective three week taper. The marked *FN14* response in MHC IIa fibers provides an initial molecular basis to support MHC IIa hypertrophy observed with taper, a stimulus that generally produces the highest calculated fast-twitch fiber growth rate in humans.

Tapered training of ≤3 weeks in aerobically fit runners [2], swimmers [1], and cyclists [3] can be calculated to confer MHC IIa growth rates of 5.0%, 8.0%, and 14.2% per week, respectively. By comparison, MHC IIa growth with progressive resistance training in young healthy individuals occurs at a maximum rate of 3.8% by 4 weeks [24] and approaches an asymptote by 12 weeks [25] as adaptation becomes more refined (see Table S1 for comprehensive review of fast-twitch growth with exercise). Despite being highly conditioned, fast-twitch muscle fibers of trained endurance athletes can rapidly change size in response to training adjustments which measurably affects whole muscle and single fiber power [1], [2], [26], [27]. However, the only transcript-level insight into mediators of fiber type-specific size regulation in humans has been in reference to resistance exercise. Acute resistance exercise elicits differential MHC I and IIa proteolytic mRNA responses [11] and activates a distinct MHC IIa transcriptome that precedes resistance training-induced increases in MHC IIa single fiber cross sectional area [6], [9]. The current investigation reinforces the differential gene regulation concept, namely that *FN14* induction may augment the capacity of MHC IIa fibers to quickly grow and improve contractile function with taper.

The 4.3-fold *FN14* induction post run in tapered MHC IIa fibers aligns with our laboratory’s recent finding that robust *FN14* expression after exercise is associated with isolated fast-twitch fiber size increases from resistance training [6], [9]. A member of the TNFα superfamily, FN14 is a cell-surface receptor found on a variety of tissues including skeletal muscle [28] and appears to signal through the diverse NF-Kβ pathway [28], [29]. While linked to atrophy in pathological conditions [30], FN14 also appears necessary for muscle proliferation, differentiation, and regeneration in mice and cell culture [31], [32]. In this investigation, *FN14* gene induction after running in the tapered state was uniform (six of six subjects) and four responses were larger (≥4.4-fold) than the largest heavily trained response (2.8-fold). These occurrences highlight the magnitude of this fiber type-specific induction and further implicate *FN14* as an important component specific to the MHC IIa remodeling process in humans’ response to exercise.

*MSTN* decreased post-run in both conditions and fiber types. Myostatin is a negative regulator of skeletal muscle mass that is blunted following a hypertrophic stimulus in humans [9], [14], [33]–[36]. Interestingly, Myostatin is also suppressed at the gene and protein level in humans after non-hypertrophic exercise [14], [37], [38]. This could signal a metabolic purpose for down-regulation [38], [39] or Myostatin’s supporting role in muscle remodeling and homeostasis after aerobic exercise [40]. It is worth noting, however, that the largest *MSTN* reduction observed here (2.0-fold) was in tapered MHC IIa fibers. Skeletal muscle reportedly has ∼2–3 times the amount of Myostatin necessary to restrain growth [41] while animal [42]–[46] and human [47]–[49] fast-twitch muscle appears most sensitive to *MSTN* alterations. The 2.0-fold tapered state reduction in *MSTN* with running may have surpassed a physiologically significant threshold for Myostatin-mediated growth to occur and could have contributed to previously observed MHC IIa hypertrophy with taper [2].

*MURF1* was responsive to run exercise in the heavily trained and tapered state in MHC IIa fibers. A marker of myofibrillar protein breakdown, *MURF1* increases after acute resistance [14], [20], [50] and endurance [14], [51] exercise and is elevated concomitant with training-induced hypertrophy [52]. Increased *MURF1* with exercise may therefore represent a normal component of the MHC IIa remodeling process in healthy human muscle. *HSP72*, *MRF4*, and *IGF1* were not altered with exercise in MHC IIa fibers in either training state. In animals, heat shock protein is less constitutively expressed in fast-twitch than slow-twitch muscle [53]–[55] and is less responsive to exercise in human Type IIa fibers [56]. A lack of *HSP72* response to intense training and taper may therefore reflect a normal pattern of heat shock protein expression in MHC IIa fibers. *MRF4* induction after taper observed previously in mixed-muscle of these runners was relatively modest (1.4 fold) [2] and was abolished at the single fiber level in both fiber types. This discrepancy could result from a combinatorial effect of all fiber types yielding significant *MRF4* expression in mixed-muscle or the potential influence of satellite cells (express *MRF4* upon activation [57], [58]). Common mRNA markers of quiescent satellite cells (*NCAM1*, *CD34*, *CDH15*, *CALCR*) are present in fibers mechanically dissected using the method described here [6] which suggests satellite cell presence but the extent of satellite cell adhesion has not yet been determined. IGF1 activates the Akt/mTOR pathway and is strongly implicated in exercise-induced muscle hypertrophy [10]. However, *IGF1* transcript is shown to not change appreciably in the early recovery from aerobic [18] or resistance exercise [18], [59], [60] which agrees with our findings.

*HSP72* responded to exercise in MHC I fibers in both training states. This induction is consistent with greater HSP72 protein levels in Type I vs IIa fibers following 30 minutes of acute exercise [56]. In well-conditioned aerobic athletes, the greatest increase in basal HSP72 protein level occurs with intensified training but overall content peaks following a reduced training period [61]. While speculative, it is conceivable that improved translational efficiency in reaction to tapering along with a slightly more pronounced *HSP72* response to exercise in tapered MHC I fibers (Figure 7) of the trained runners studied here ultimately results in greater HSP72 protein levels after taper. This could be interpreted as a positive adjustment to the post run cellular environment that would aid in the localized stress tolerance of intense exercise performance [12] that coincides with peak level competition [1], [2], [4].

A properly conducted taper program in athletes prior to peak competition improves performance that appears to be mediated, in part, by increased power at the whole muscle and single muscle fiber level. The single muscle fiber performance gains with taper are targeted to MHC IIa fibers and are primarily driven by hypertrophy [1], [2]. The current study extends these MHC IIa-specific muscle fiber adaptations with taper to the molecular level and reports a sizeable *FN14* induction in the tapered state that may have contributed to rapid fast-twitch remodeling. A potentially meaningful *MSTN* suppression with tapered state exercise may also help support a favorable fast-twitch growth environment. We speculate that intense aerobic training periods reduce MHC IIa plasticity of growth-related gene targets and that taper reverses this process. To what extent the taper-induced MHC IIa adaptations are due to super-compensation from intense training or recovery to levels prior to the heavy training phase has not been established. The current study illustrates how tapering can promote transcriptional flexibility not previously thought possible in well-conditioned muscle [6], [8]. These basic alterations at the molecular level provide a framework for expanded studies (i.e. transcriptome, protein quantification, epigenetics, etc.) to further our understanding of MHC IIa plasticity with exercise training paradigms.

## Supporting Information

Table S1 Summary of literature on selective fast-twitch size alterations in young healthy individuals.
(PDF)
