# Supplementary material for: Single Muscle Fiber Gene Expression with Run Taper
Source: PLoS One. 2014 Sep 30;9(9):e108547. doi: 10.1371/journal.pone.0108547 (PMC4182496; doi:10.1371/journal.pone.0108547)
Supplement: Table S1 — Summary of literature on selective fast-twitch size alterations in young healthy individuals. (PDF) [file pone.0108547.s001.pdf]

Table S1. Summary of literature on selective fast-twitch size alterations in young healthy individuals.

Reference	Subject Population	Training Stimulus	Sex (n)	Age (y)	MHC IIa CSA (%Δ)	Duration	Rate (%Δ/week)	Muscle	Method
Resistance Training									
Hakkinen 1981 (17)	Recreational	Con/Ecc	M (14)	20-30	↑ 13.5%[a]	8 wks[b]	↑ 1.7%	VL	H
Houston 1983 (25)	Recreational	Con/Ecc	M (6)	20±1	↑ 21.0%	10 wks	↑ 2.1%	VL	H
Hakkinen 1985 (16)	Recreational	Con/Ecc	M (11)	20-32	↑ 28.8%[a]	24 wks	↑1.2%	VL	H
Tesch 1987 (38)	Recreational	Con/Ecc/Ecc and Explosive	M (11, 10)	26±4, 27±3	↑ 28.9%, 12.8%	24 wks	↑1.2%, 0.5%	VL	H
Alway 1989 (2)	Untrained	Isometric	M (7)	25±3	↑ 50.0%	16 wks	↑ 3.1%	LG	H
Alway 1990 (3)	Untrained	Isometric	M (6)	27±3	↑ 30.0%	16 wks	↑ 1.9%	LG	H
Staron 1991 (37)	Untrained	High-Intensity Retraining	F (7)	21±1	↑ 18.0%	6 wks	↑ 3.0%	VL	H
Hather 1991 (20)	Recreational	High-Volume Concentric	M (10)	33±1	↑ 27.0%	19 wks	↑ 1.4%	VL	H
Hickson 1994 (21)	Recreational	Con/Ecc	M (5) + F (5)	29±1	↑ 19.0%	16 wks	↑ 1.2%	VL	H
Hortobagyi 1996 (22)	Untrained	Eccentric Overload	M (7)	20±1	↑ 37.8%	12 wks	↑ 3.2%	VL	H
Volek 1999 (40)	Trained	Con/Ecc	M (9)	25±6	↑ 5.9%	12 wks	↑ 0.5%	VL	H
Andersen 2000 (4)	Untrained	Con/Ecc	M (9)	27±3	↑ 18.6%	12 wks	↑ 1.6%	VL	H
Aagaard 2001 (1)	Untrained	Con/Ecc	M (11)	27±5	↑ 18.4	12 wks	↑ 1.5%	VL	H
Harber 2004 (18)	Untrained	Circuit Training	M (8)	24±2	↑ 21.2%	10 wks	↑ 2.1%	VL	H
Cribb 2006 (11)	Recreational Bodybuilders	Con/Ecc with PRO/CP	M (8)	21±3	↑ 25.0%	10 wks	↑ 2.5%	VL	H
Woolstenhulme 2006 (41)	Untrained	Con/Ecc	M (6)	21±4	↑ 15.1%	4 wks (8 wks total)	↑ 3.8%	VL	H
Cribb 2007 (12)	Recreational Bodybuilders	Con/Ecc with CHO	M (7)	24±7	↑ 4.8%	10 wks	↑ 0.5%	VL	H
Hartman 2007 (19)	Untrained	Con/Ecc with CHO	M (19)	18-30	↑ 11.8%	12 wks	↑ 1.0%	VL	H
Raue 2009 (34)	Untrained	Con/Ecc	F (9)	21±2	↑ 28.0%	12 wks	↑ 2.3%	VL	SFD
Claflin 2011 (10)	Untrained	Con/Ecc and High Velocity	M (15) + F (18)	25±--	↑ 8.3%	14 wks	↑ 0.6%	VL	SFD
Farup 2012 (13)	Untrained	Con/Ecc	M (18)	23±1	↑ 22.0%	10 wks	↑ 2.2%	VL	H
Schuenke 2012 (36)	Untrained	Low Velocity Con/Ecc	F (10)	19±1	↑ 13.0%	6 wks	↑ 2.2%	VL	H
Aerobic Training									
Andersen 1977 (5)	Untrained	Cycling	M (5)	20-23	↑ 16.8%	8 wks	↑ 2.1%	VL	H
Houston 1979 (24)	Trained	Run Training After Detraining	M (3)	33±3	↑ 32.4%[cd]	15 d	↑ 15.1%	LG	H
Bangsbo 1988 (6)	Semi-Pro Soccer Players	General Conditioning After Detraining	M (4)	23±--	↑ 16.3%[d]	4 wks	↑ 4.1%	LG	H
Mizuno 1990 (31)	Trained	X-Country Skiing at Altitude	M (10)	22±--	↑ 4.3%	2 wks	↑ 2.2%	TB	H

Table S1 (continued). Summary of literature on selective fast-twitch size alterations in young healthy individuals.

Reference	Subject Population	Training Stimulus	Sex (n)	Age (y)	MHC IIa CSA (%Δ)	Duration	Rate (%Δ/week)	Muscle	Method
Combined Training									
Kraemer 1995 (27)	Recreational	Con/Ecc + Running	M (9)	23±4	↑ 20.1%	12 wks	↑ 1.7%	VL	H
Bell 2000 (7)	Recreational	Con/Ecc + Cycling	M (8) + F (5)	22±3	↑ 13.8%	12 wks	↑ 1.2%	VL	H
Chilibeck 2002 (9)	Untrained	Con/Ecc + Cycling	M (5) + F (5)	24±3	↑ 17.6%[a]	12 wks	↑ 1.5%	VL	H
McCarthy 2002 (30)	Untrained	Con/Ecc + Cycling	M (10)	27±2	↑ 27.8%	10 wks	↑ 2.8%	VL	H
Putman 2004 (33)	---	Con/Ecc + Cycling	[M + F] (10)	22±--	↑ 18.0%	12 wks	↑ 1.5%	VL	H
Lundberg 2013 (29)	Recreational	Con/Ecc + Cycling	M (10)	25±4	↑ 18.8%	5 wks	↑ 3.4%	VL	H
Intensified Training									
Fitts 1989 (14)	Trained	Swimming	M (12)	19±--	↓ 32.6%	10 d	↓ 22.8%	Deltoid	SFD
Kohn 2011 (26)	Trained	Running	M (18)	---	↓ 17.0%[e]	6 wks	↓ 2.8%	VL	H
Taper									
Trappe 2000 (39)	Trained	Swimming	M (6)	20±1	↑ 24.0%	3 wks	↑ 8.0%	Deltoid	SFD
Neary 2003 (32)	Trained	Cycling	M (22)	25±6	↑ 14.2%	7 d	↑ 14.2%	VL	H
Luden 2010 (28)	Trained	Running	M (7)	20±1	↑ 15.0%	3 wks	↑ 5.0%	LG	SFD
Detraining									
Houston 1979 (24)	Trained	Running	M (3)	33±3	↑ 24.3%[c]	15 d	↑ 11.3%	LG	H
Schantz 1983 (35)	Recreational	Weighted Skiing	M (6)	25±--	↓ 20.0%	27 wks	↓ 0.7%	TB	H
Bangsbo 1988 (6)	Semi-Pro Soccer Players	General Conditioning	M (4)	23±--	↓ 14.1%	3 wks	↓ 4.7%	LG	H
Hortobagyi 1993 (23)	Football Players	Power Activity	M (12)	24±1	↓ 6.4%	2 wks	↓ 3.2%	VL	H
Billeter 2003 (8)	Olympic Champion Shot Putter	Power Activity	M (1)	32	↓ 24.5%[c]	3 yrs	---	VL	H
Gjovaag 2008 (15)	Untrained	Con/Ecc	M (12) + F (20)	22±1	↓10.1%[f]	8 wks	↓ 1.3%	TB	H

[a]=Estimated from figure, [b]=of a 16 wk study, [c]=Non-statisical due to low *n* but larger than slow-twitch Δ, [d]=Compared to trained baseline, [e]=P=0.06, [f]=Compared to post-training, CON/ECC=Concentric and Eccentric Resistance Training, VL=Vastus Lateralis, LG=Lateral Gastrocnemius, TB=Triceps Brachii, H=Histochemistry, SFD=Single Fiber Diameter, PRO/CP=Protein/Creatine Supplementation, CHO=Carbohydrate Supplementation

References

1. Aagaard P, Andersen JL, Dyhre-Poulsen P, Leffers AM, Wagner A, et al. (2001) A mechanism for increased contractile strength of human pennate muscle in response to strength training: changes in muscle architecture. J Physiol 534: 613-623.
2. Alway SE, MacDougall JD, Sale DG (1989) Contractile adaptations in the human triceps surae after isometric exercise. J Appl Physiol 66: 2725-2732.
3. Alway SE, Sale DG, MacDougall JD (1990) Twitch contractile adaptations are not dependent on the intensity of isometric exercise in the human triceps surae. Eur J Appl Physiol Occup Physiol 60: 346-352.
4. Andersen JL, Aagaard P (2000) Myosin heavy chain IIX overshoot in human skeletal muscle. Muscle Nerve 23: 1095-1104.
5. Andersen P, Henriksson J (1977) Capillary supply of the quadriceps femoris muscle of man: adaptive response to exercise. J Physiol 270: 677-690.
6. Bangsbo J, Mizuno M (1988) Morphological and metabolic alterations in soccer players with detraining and retraining and their relation to performance. Science and Football: 114-124.
7. Bell GJ, Syrotuik D, Martin TP, Burnham R, Quinney HA (2000) Effect of concurrent strength and endurance training on skeletal muscle properties and hormone concentrations in humans. Eur J Appl Physiol 81: 418-427.
8. Billeter R, Jostarndt-Fogen K, Gunthor W, Hoppeler H (2003) Fiber type characteristics and myosin light chain expression in a world champion shot putter. Int J Sports Med 24: 203-207.
9. Chilibeck PD, Syrotuik DG, Bell GJ (2002) The effect of concurrent endurance and strength training on quantitative estimates of subsarcolemmal and intermyofibrillar mitochondria. Int J Sports Med 23: 33-39.
10. Claflin DR, Larkin LM, Cederna PS, Horowitz JF, Alexander NB, et al. (2011) Effects of high- and low-velocity resistance training on the contractile properties of skeletal muscle fibers from young and older humans. J Appl Physiol 111: 1021-1030.
11. Cribb PJ, Hayes A (2006) Effects of supplement timing and resistance exercise on skeletal muscle hypertrophy. Med Sci Sports Exerc 38: 1918-1925.
12. Cribb PJ, Williams AD, Stathis CG, Carey MF, Hayes A (2007) Effects of whey isolate, creatine, and resistance training on muscle hypertrophy. Med Sci Sports Exerc 39: 298-307.
13. Farup J, Kjolhede T, Sorensen H, Dalgas U, Moller AB, et al. (2012) Muscle morphological and strength adaptations to endurance vs. resistance training. J Strength Cond Res 26: 398-407.
14. Fitts RH, Costill DL, Gardetto PR (1989) Effect of swim exercise training on human muscle fiber function. J Appl Physiol 66: 465-475.
15. Gjovaag TF, Dahl HA (2008) Effect of training with different intensities and volumes on muscle fibre enzyme activity and cross sectional area in the m. triceps brachii. Eur J Appl Physiol 103: 399-409.

16. Hakkinen K, Alen M, Komi PV (1985) Changes in isometric force- and relaxation-time, electromyographic and muscle fibre characteristics of human skeletal muscle during strength training and detraining. Acta Physiol Scand 125: 573-585.
17. Hakkinen K, Komi P, Tesch P (1981) Effect of combined concentric and eccentric strength training and detraining on force-time, muscle fiber and metabolic characteristics of leg extensor muscles. Scand J Sports Sci 3: 50-58.
18. Harber MP, Fry AC, Rubin MR, Smith JC, Weiss LW (2004) Skeletal muscle and hormonal adaptations to circuit weight training in untrained men. Scand J Med Sci Sports 14: 176-185.
19. Hartman JW, Tang JE, Wilkinson SB, Tarnopolsky MA, Lawrence RL, et al. (2007) Consumption of fat-free fluid milk after resistance exercise promotes greater lean mass accretion than does consumption of soy or carbohydrate in young, novice, male weightlifters. Am J Clin Nutr 86: 373-381.
20. Hather BM, Tesch PA, Buchanan P, Dudley GA (1991) Influence of eccentric actions on skeletal muscle adaptations to resistance training. Acta Physiol Scand 143: 177-185.
21. Hickson RC, Hidaka K, Foster C, Falduto MT, Chatterton RT, Jr. (1994) Successive time courses of strength development and steroid hormone responses to heavy-resistance training. J Appl Physiol 76: 663-670.
22. Hortobagyi T, Hill JP, Houmard JA, Fraser DD, Lambert NJ, et al. (1996) Adaptive responses to muscle lengthening and shortening in humans. J Appl Physiol 80: 765-772.
23. Hortobagyi T, Houmard JA, Stevenson JR, Fraser DD, Johns RA, et al. (1993) The effects of detraining on power athletes. Med Sci Sports Exerc 25: 929-935.
24. Houston ME, Bentzen H, Larsen H (1979) Interrelationships between skeletal muscle adaptations and performance as studied by detraining and retraining. Acta Physiol Scand 105: 163-170.
25. Houston ME, Froese EA, Valeriote SP, Green HJ, Ranney DA (1983) Muscle performance, morphology and metabolic capacity during strength training and detraining: a one leg model. Eur J Appl Physiol Occup Physiol 51: 25-35.
26. Kohn TA, Essen-Gustavsson B, Myburgh KH (2011) Specific muscle adaptations in type II fibers after high-intensity interval training of well-trained runners. Scand J Med Sci Sports 21: 765-772.
27. Kraemer WJ, Patton JF, Gordon SE, Harman EA, Deschenes MR, et al. (1995) Compatibility of high-intensity strength and endurance training on hormonal and skeletal muscle adaptations. J Appl Physiol 78: 976-989.
28. Luden N, Hayes E, Galpin A, Minchev K, Jemiolo B, et al. (2010) Myocellular basis for tapering in competitive distance runners. J Appl Physiol 108: 1501-1509.

29. Lundberg TR, Fernandez-Gonzalo R, Gustafsson T, Tesch PA (2013) Aerobic exercise does not compromise muscle hypertrophy response to short-term resistance training. J Appl Physiol 114: 81-89.
30. McCarthy JP, Pozniak MA, Agre JC (2002) Neuromuscular adaptations to concurrent strength and endurance training. Med Sci Sports Exerc 34: 511-519.
31. Mizuno M, Juel C, Bro-Rasmussen T, Mygind E, Schibye B, et al. (1990) Limb skeletal muscle adaptation in athletes after training at altitude. J Appl Physiol 68: 496-502.
32. Neary JP, Martin TP, Quinney HA (2003) Effects of taper on endurance cycling capacity and single muscle fiber properties. Med Sci Sports Exerc 35: 1875-1881.
33. Putman CT, Xu X, Gillies E, MacLean IM, Bell GJ (2004) Effects of strength, endurance and combined training on myosin heavy chain content and fibre-type distribution in humans. Eur J Appl Physiol 92: 376-384.
34. Raue U, Slivka D, Minchev K, Trappe S (2009) Improvements in whole muscle and myocellular function are limited with high-intensity resistance training in octogenarian women. J Appl Physiol 106: 1611-1617.
35. Schantz P, Henriksson J, Jansson E (1983) Adaptation of human skeletal muscle to endurance training of long duration. Clin Physiol 3: 141-151.
36. Schuenke MD, Herman JR, Gliders RM, Hagerman FC, Hikida RS, et al. (2012) Early-phase muscular adaptations in response to slow-speed versus traditional resistance-training regimens. Eur J Appl Physiol 112: 3585-3595.
37. Staron RS, Leonardi MJ, Karapondo DL, Malicky ES, Falkel JE, et al. (1991) Strength and skeletal muscle adaptations in heavy-resistance-trained women after detraining and retraining. J Appl Physiol 70: 631-640.
38. Tesch PA, Komi PV, Hakkinen K (1987) Enzymatic adaptations consequent to long-term strength training. Int J Sports Med 8 Suppl 1: 66-69.
39. Trappe S, Costill D, Thomas R (2000) Effect of swim taper on whole muscle and single muscle fiber contractile properties. Med Sci Sports Exerc 32: 48-56.
40. Volek JS, Duncan ND, Mazzetti SA, Staron RS, Putukian M, et al. (1999) Performance and muscle fiber adaptations to creatine supplementation and heavy resistance training. Med Sci Sports Exerc 31: 1147-1156.
41. Woolstenhulme MT, Conlee RK, Drummond MJ, Stites AW, Parcell AC (2006) Temporal response of desmin and dystrophin proteins to progressive resistance exercise in human skeletal muscle. J Appl Physiol 100: 1876-1882.
